# Advanced delivery of natural bioactive compounds for atopic dermatitis: a review of polymeric conjugates and nanocarriers

**DOI:** 10.1007/s13659-025-00585-w

**Published:** 2026-02-05

**Authors:** Jigar Vyas, Nensi Raytthatha, Puja Vyas, Sudarshan Singh, Bhupendra G. Prajapati

**Affiliations:** 1 Krishna School of Pharmacy and Research, Drs. Kiran and Pallavi Global University, Varnama, Vadodara, 391240 Gujarat India; 2 Sigma Institute of Pharmacy, Sigma University, Vadodara, 390019 Gujarat India; 3 Office of Research Administration, Chiang Mai University, Chaing Mai, 50200 Thailand; 4 Faculty of Pharmacy, Chiang Mai University, Chaing Mai, 50200 Thailand; 5 Department of Pharmaceutics, Faculty of Pharmacy, Parul Institute of Pharmacy, Parul University, Waghodia, Vadodara, 391760 Gujarat India; 6 Centre for Research Impact & Outcome, Chitkara College of Pharmacy, Chitkara University, Rajpura, 140401 Punjab India

**Keywords:** Atopic dermatitis, Bioactive compounds, Polymeric conjugates, Nanocarriers, Skin barrier, Anti-inflammatory therapy, Herbal formulations

## Abstract

Atopic dermatitis (AD) is a persistent, relapsing skin inflammatory disorder characterised by epidermal barrier dysfunction, immune system anomalies, and microbial dysbiosis. Current therapies encompass calcineurin inhibitors, topical corticosteroids, and synthetic drugs such as JAK inhibitors and biologics, which, despite their efficacy, have safety issues and constraints on prolonged use. Recently, natural bioactive compounds namely flavonoids, polyphenols, and terpenoids have garnered interest for their anti-inflammatory, antioxidant, and skin-barrier-repairing characteristics. Recent studies emphasise the incorporation of these bioactive compounds into hydrophilic/hydrophobic polymeric conjugates and nanocarrier systems to promote skin distribution, increase efficacy, and minimise systemic adverse effects. This study encapsulates traditional and innovative medicines, highlights the promise of polymer-conjugated herbal formulations, and examines recent developments in nanotechnology and delivery technologies for AD management. The increasing interest in bioactive ingredient formulations and polymeric nanocarriers indicates a favourable shift towards safer, more effective, and patient-compliant treatment methodologies.

**Graphical Abstract:**

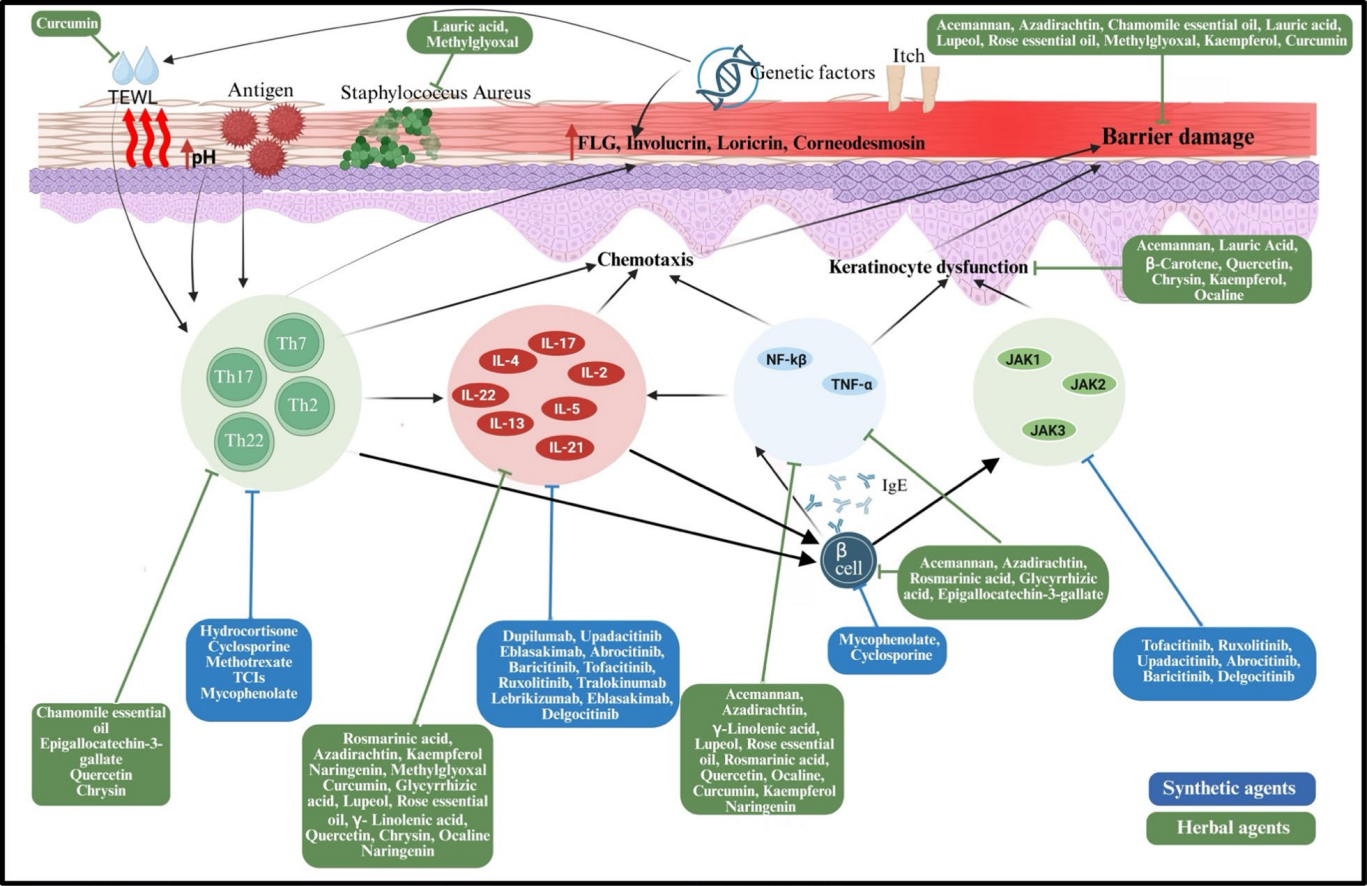

## Introduction

Skin inflammation is the physiologic response to harmful stimuli such as pathogens, irritants, allergens, trauma, or autoimmune mediators; and is manifested by erythema, heat, edema, pain, and possibly function loss [1–3]. It is utilized to remove the cause of injury, remove damaged tissue and to begin the healing process. However, when chronic or dysregulated, inflammation can lead to tissue damage and contribute to a number of skin disorders [4]. On the basis of time period and pathology, it is classified into acute or chronic. Acute inflammation is brief and is marked by vascular changes, neutrophil infiltration and mediator release in order to resolve the injury, as is seen in contact dermatitis or insect bites [5]. Chronic inflammation on the other hand is characterized by the presence of persistent or unresolved stimuli and is associated with immune cell transitions towards lymphocytes and macrophages with fibrotic and tissue remodelling [6, 7]. This state is characteristic of chronic dermatoses such as psoriasis, lichen planus, and AD, which are characterized by immune dysregulation that results in de-structuring of normal skin and impairment of the barrier function [8, 9].

AD is a still important public health problem because of its chronic relapsing course, psychosocial burden and because of the limitations of current pharmacotherapies. Although topical corticosteroids, calcineurin inhibitors, biologics, and JAK inhibitors are clinically efficacious, long-term safety concerns, high cost, and incomplete therapeutic response have led to a paradigm shift in this multifactorial disease to natural bioactive compounds with multi-targeted effects. However, these compounds have low solubility, stability, and dermal penetration which limits their clinical translation. To circumvent these obstacles, innovative hydrophilic/hydrophobic polymeric conjugates and delivery systems based on nanocarriers have emerged as new promising tools to improve skin retention, controlled release and avoid adverse systemic side effects, an aspect that constitutes the main part of this review [10, 11].

### Pathogenesis of atopic dermatitis

**Fig. 1:**
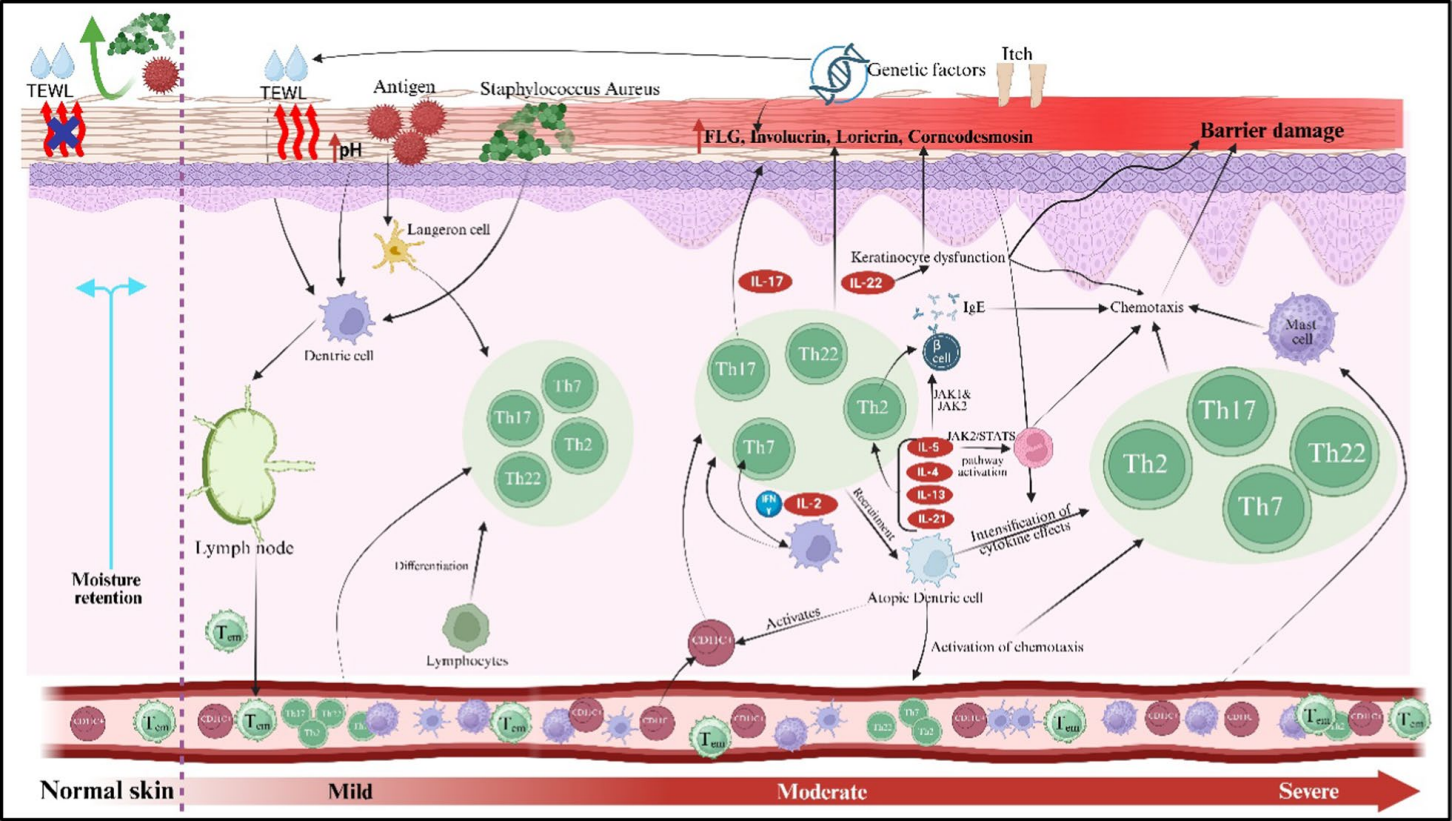
Pathogenesis of atopic dermatitis

The pathogenesis, as depicted in Fig. 1, involves a interplay of genetic, immunological, and ecological variables. The core condition is epidermal barrier malfunction, predominantly resulting from mutation in the FLG (filaggrin) gene, which compromises skin hydration and integrity, hence increasing trans epidermal water loss (TEWL) and facilitating allergy and microbial penetration. Further changes in barrier proteins such as claudin-1 and involucrin impair tight junctions, hence weakening the protective capabilities of skin. Immune dysregulation in AD is primarily influenced by a predominant response of Th2. Following barrier disruption, dendritic and Langerhans cells exhibit antigens, facilitating Th2 differentiation. These cells produce IL4, IL5, IL13, and IL31, which stimulate IgE synthesis, eosinophil recruitment, and pruritus, hence sustaining the itch-scratch cycle. As AD advances, additional T-helper subsets, including Th22, Th17, and Th1, also play their role; for instance, IL22 facilitates epidermal thickening, but IL17 and IFN-γ exacerbate inflammation and compromise barrier integrity, illustrating variability in AD across age as well as phenotype. Microbial dysbiosis, especially the colonisation by Staphylococcus aureus, intensifies inflammation through superantigens and toxins while displacing beneficial commensals. The neuroimmune axis, via IL31 and neuropeptides, activates sensory neurones, exacerbating pruritus and perpetuating chronic inflammation through the itch-scratch-injury cycle [12–14].

The clinical phenotypes of AD show considerable variability based on age, severity, and ethnicity, indicating a complex interaction of characteristics that may offer avenues for preventative and therapeutic strategies [15]. AD severity is categorised into three levels: mild, moderate, and severe. People with AD can be assigned to four groups based on when their first symptoms: teenage, childhood, early onset, very early onset. and. AD is further divided into mild, moderate, and severe based on the severity. In most cases, mild AD indicates as small, dry, itchy plaque that sometimes turn red and causes little interference with daily activities. Moderate AD presents with more widespread skin involvement, more intense and persistent itching, redness, and sometimes skin thickening (lichenification) due to chronic scratching and severe AD is the most debilitating form, marked by extensive areas of inflamed, thickened, and sometimes oozing or crusted skin. Patients often endure persistent pruritus, recurrent outbreaks, and a lower quality of life. This condition is typically linked to sleep difficulties, psychological strain, and a higher incidence for other skin infections. In 89% of patients, pruritus remains the predominant symptom and the early manifestation of AD, leading to infections, discomfort, anxiety, disruption of the epidermal skin layer, and sleeplessness [16]. Children, adolescents, and adults have diffused flexural plaques that fluctuate between acute and chronic phases, unlike infants, who present with acute eczematous pruritic lesions affecting the cheeks, face, and torso [17].

Differentiating AD from other dermatological diseases is important in older patients. Initial signs are reminiscent of psoriasis, erythroderma, scabies, or cutaneous T cell lymphoma. Disease severity is generally assessed by validated scoring systems including the SCORAD (SCORing Atopic Dermatitis) and EASI (Eczema Area and Severity Index), which measure clinical signs such as erythema, oedema, excoriations, lichenification and area of lesion extension with crusted or wet aspect of lesions SCORAD also includes weeping and crusts as markers of active disease. A holistic comprehension of the multilateral pathology of AD immune discoordination, epidermal barrier failure, and microbial incongruence is essential for devising an effective therapeutic approach [18].

### Management of atopic dermatitis

The therapeutic management encompasses a broad spectrum of approaches, ranging from conventional treatments to advanced biologics. While conventional therapies remain the cornerstone for symptom control and flare management, recent advancements in targeted immunomodulatory drugs have changed the treatment landscape, particularly for severe to moderate cases.

### Synthetic agents of atopic dermatitis and new alternatives

Traditional treatment of AD falls into two categories of topical and systemic treatment depending on the severity of the disease. Topical corticosteroids (TCSs) are still used as the first line of treatment of acute flares and mild-to-moderate AD. They prevent the occurrence of local inflammation by blocking the production of cytokines and T-cell activation. The level of potency is mild (hydrocortisone) to very potent (clobetasol propionate) and the choice of one relies on the location of the lesion, age, and the extent of the disease. Long-term use is however not without side effects that include skin atrophy, telangiectasis, and perioral dermatitis hence the intermittent and site-specific application [19].

Topical calcineurin inhibitors (TCIs), which include pimecrolimus (Elidel(r)) and tacrolimus (Protopic(r)) are non-steroid immunomodulators used to reduce the involvement of steroid-associated toxicity by blocking the activation of T- cells and release of IL-2 through calcium signalling. These are preferred as they are used in maintenance therapy where they prevent the flares without causing skin thinning [20, 21]. In moderate-severe or refractory cases, systemic immunosuppressants such as cyclosporine, methotrexate, azathioprine and mycophenolate mofetil are employed. Cyclosporine is fast acting and is restricted by nephrotoxicity and hypertension. Close observation is necessary in other agents because they have hepatotoxicity or bone marrow suppression. Antihistamines can be given orally as an addition, such as diphenhydramine and hydroxyzine, to act as an adjunct to relieve pruritus and sleep; however, they do not possess anti-inflammatory activity.

In view of the toxicity and pharmacokinetic profile of the conventional immunosuppressants, the targeted synthetic therapies represented by mAbs and JAK inhibitor mark a major milestone in the history of AD treatment. The first biologic approved for use by FDA and EMA is dupilumab, which blocks IL-4 and IL-13 by blocking IL-4Ra, reaching highly significant suppression of inflammation and pruritus with a very good safety profile. Side effects of interest are mainly conjunctivitis. Further IL-13-blocking mAbs are tralokinumab, lebrikizumab, and eblasakimab, which block IL-13-signaling by different receptor-binding mechanisms offering further specific cytokine blockade [22, 23].

JAK inhibitors are the next generation of oral small-molecule therapy which disrupts cytokine-mediated (IL-4, IL-13, IL-31, IFN-g) signals via the JAK -STAT pathway. Acceptable ones are upadacitinib and abrocitinib (JAK1-selective) and baricitinib (JAK1/2-inhibitor). These agents have a quick onset and high reduction in the severity of the disease, but they have the class-related risks of infections, thrombosis, and lipid elevation. Topical therapies such as ruxolitinib (JAK1/2) and delgocitinib (pan-JAK) are less toxic than systemic JAK inhibitors, and can be used as topical agents with localized effects [24–26]. Although synthetic therapies have demonstrated clinical effectiveness, there exist significant limitations such as; systemic toxicity, risk of infection, high cost, and unavailability, especially in low-resource communities. Corticosteroids, calcium-inhibitor, or JAK inhibitors intensify the use of long-term corticosteroids, induce irritation or malignancy-related issues or necessitate continued safety surveillance, respectively. Also, the majority of synthetic agents act on individual molecular pathways, which is insufficient to consider the multifactorial pathogenesis of AD related to immune dysregulation, dysfunction of the barrier, and microbial imbalance [27]. As a result, herbal and plant-based treatment is receiving attention as complementary or alternative ways of treatment. Multi-targeted activity like anti-inflammatory, antioxidant, antimicrobial and barrier-restorative, medicinal herbs, including turmeric, liquorice, aloe vera and neem, provide symptomatic relief that has lesser systemic side effects. Herbal strategies exhibit a bright future in the complex treatment of AD, though additional clinical confirmation and standardization is necessary, herbal methods offer an alternative and accessible way to supplement synthetic medications and a safer treatment option.

### The use of herbal and bioactive compounds in management of atopic dermatitis

**Fig. 2:**
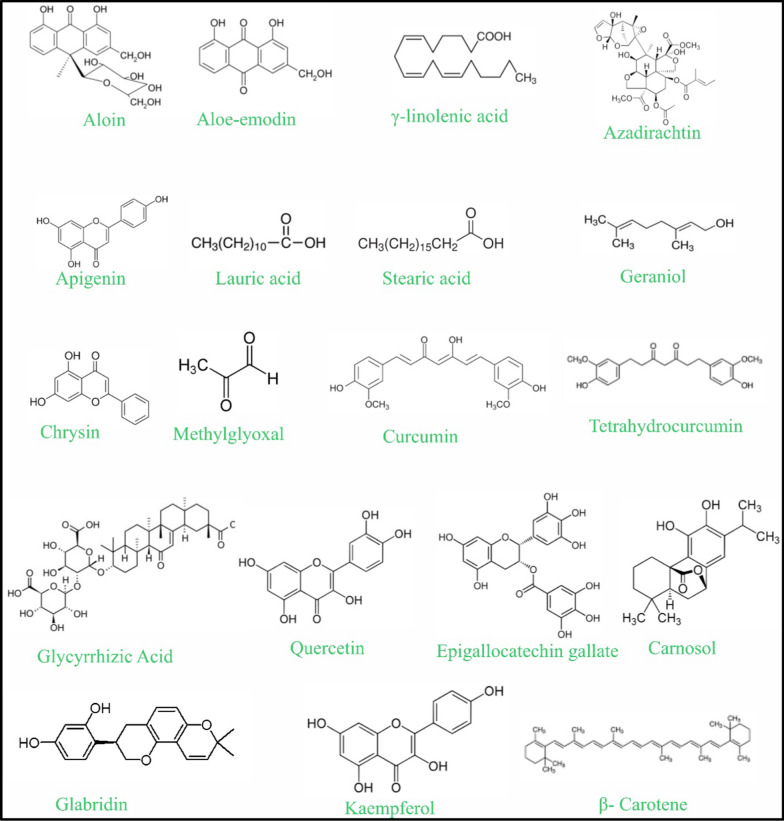
Chemical structures of key bioactive compounds identified from herbal agents used in the management of atopic dermatitis

Plant and herbal therapies may serve as safe and effective alternatives or adjuncts to current synthetic medications for AD, given their diverse effects, including anti-inflammatory, antimicrobial, antioxidant, and immunomodulatory properties, alongside low systemic toxicity. These agents as shown in Fig. 2 may contain high levels of bioactive molecules like flavonoids, terpenoids, polyphenols, and essential fatty acids that control cytokine signalling, stop mast cell degranulation, and restore skin barrier function, which are the main causes of AD [28].

The anti-inflammatory, wound-healing, and immunomodulatory properties of compounds such as aloin and aloe-emodin in aloe vera, one of the oldest known medicinal plants, suppress the production of inflammatory cytokines and bacteria growth and repair damaged skin [29, 30]. Similarly, Borage seed oil (Borago officinalis) is a good source of g-linolenic acid (GLA) and linoleic acid that can restore missing fatty acids in AD patients, enhance the condition of dryness, itchiness, and inflammation by restoring eicosanoid [31, 32].

Neem (Azadirachta indica), an Ayurvedic medicine, contains nimbidin, nimbolide, and azadirachtin, which repress the macrophage and neutrophil activation and production of pro-inflammatory cytokines that include IL-1β and TNF-α [33]. Chamomile (Matricaria recutita) has flavonoids and terpenoids; it is used to treat the inflamed skin by its volatile oils, which infiltrate deep into the skin up to the dermis layer, preventing irritation and erythema [34]. Coconut oil, which is rich in lauric, myristic, and linoleic acids, enhances barrier strength concerning trans-epidermal water loss prevention together with high antimicrobial efficiency against Staphylococcus aureus. In the same vein, triterpenes, tocophenols, and phenolic compounds present in Shea butter (Vitellaria paradoxa) suppress IL and cytokines production, thus enhancing hydration and reducing inflammation [35].

Plant-derived essential oils also demonstrate substantial therapeutic potential. For example, rose oil (Rosa spp.), which contains bioactive constituents such as quercetin, kaempferol, anthocyanins, and phenolic acids, has been shown to attenuate NF-κB, COX-2, and iNOS signalling pathways, thereby reducing the production of pro-inflammatory cytokines (IL-1β, IL-6, and TNF-α) [36]. Additionally, its antioxidant properties help preserve epidermal barrier integrity and support tissue repair. Anti-inflammatory activity, rosmarinic acid, carnosic acid and ursolic acid which are present in Rosemary (Rosmarinus officinalis), prevents anti-TLR2-mediated and anti-NF-kB-mediated signals, reducing the expression of IL-1b, IL-8 and TNF-a, and has anti-inflammatory effects in vivo and in vitro [37]. Honey is a natural combination of flavonoids and phenolic acids, which include chrysin, which is known to mediate immune responses by inhibiting NF-kB and MAPK signalling, Th1/Th2/Th17 cytokines, and mast infiltration and release of histamine [38].

In the case of isolated phytoconstituents, quercetin, which is abundant in citrus peels and onions, has potent anti-inflammatory and antioxidant effects by inhibiting NF-kB, MAPK and AP-1 pathways, by reducing ROS-mediated keratinocyte apoptosis, and by inhibiting IL-4, IL-5 and TNF-α. Topical quercetin preparations, e.g., Quercevita(r), increase hydration and decrease pruritus with new liposomal and nanoparticle-based systems showing an improved penetration rate of the compound into the skin [39]. The green tea epigallocatechin gallate (EGCG) inhibits T- and B- cell migration, histamine release and pro-inflammatory cytokines (TNF-a, IFN-g, IL-2), and stimulates fibroblast survival and collagen synthesis. Microneedle patches and nanoparticles are advanced carriers, which have increased its stability and retinene in the dermis [40, 41].

Other flavonoid including kaempferol and naringenin also provide evidence on efficacy on AD models. Kaempferol [42] is able to inhibit JNK and NF-kB signaling, lower IL-4 and IL-13-production, reduce TEWL, and replenish barrier proteins. The citrus fruit naringenin down regulates NF-kB signaling, decreasing TNF-a, IL-6 and iNOS expression and experimental liposomal preparations exhibit greater penetration into the skin. b-carotene, a vitamin A precursor, also has antioxidant and anti-inflammatory effects [43].

Commercial phyto-complexes such as Ocalinetm PF, which is a combination of marine spring water and Cucurbita pepo seed extract have demonstrated clinical potentials in inhibiting the release of substance P (IL-8, TNF-a), in terms of neurogenic inflammation and skin sensitivity. Even though there is a lot of promise with herbal and bioactive compounds, their application in clinical settings is still minimal due to low solubility, instability, and low skin permeability [44]. To address such limitations, polymeric, liposomal, and nanoparticle delivery systems are under development to overcome barriers of dermal absorption, sustained release and improved bioavailability. Combining nanotechnology and phytopharmacology therefore is a critical milestone in the design of effective, safe and standardized plant-based formulations in chronic and relapsing AD. Table 1 lists important herbal agents, their active ingredients, polymer-based formulations that go along with them, and how they work in preclinical and clinical models of atopic dermatitis.

**Table 1:** Details of herbal and bioactive compounds used for the treatment of atopic dermatitis

Herb(s)	Bioactive compound(s)	Polymer and formulation	Mechanism of Anti-AD effect	Key findings	Ref
*Curcuma longa*	Tetrahydro-curcumin	Solid lipid nanoparticle-based gel	Enhanced skin penetration, significant reduction in TNF-α and IL6, complete lesion healing in 2,4-dinitrochlorobenzene-induced (DNCB)) AD mouse model	SLN formulation improved skin penetration and provided faster lesion resolution compared to non-encapsulated drug	[45]
*Curcuma longa*	Curcumin	Intraperitoneal injection (20 mg/kg/day)	In a BALB/c mouse model of ovalbumin-induced AD, curcumin significantly reduced epidermal thickening and inflammatory cell infiltration	Demonstrated strong systemic immunomodulatory effects. This suggests CUR's strong immunomodulatory and anti-inflammatory effect in systemic administration for AD	[46]
*Curcuma longa*	Curcumin	Topical polyherbal cream (Herbavate^®^)	Clinical improvement in eczema symptoms (itching, erythema, thickening, flaking) after 4 weeks of topical application twice daily in 150 patients. However, limitations include no control group, potential CUR-induced contact dermatitis, and dropouts that affect interpretability	Improvement in eczema symptoms after 4 weeks	[47]
*Curcuma longa*	Curcumin Turmeric rhizome extract (1%)	Moisturizing nanoemulgel	Application significantly reduced AD-like symptoms and levels of TSLP, IL13, IL17, and histopathological scores in a DNCB-induced model. However, its effect on TEWL was not statistically significant. Effective in reducing inflammation and lesion severity	Nano-emulgel improved drug dispersion and skin absorption	[48]
*Camellia sinesis*	Epigallocatechin-3-gallate (EGCG)	Poly-γ-glutamate microneedle hydrogel patch	Sustained EGCG release, antioxidant and immunomodulatory effects; reduced IgE, histamine, Th2 cytokines in Nc/Nga mice	Microneedles enabled controlled transdermal delivery and prolonged therapeutic effect with lower dosing	[49]
*Glycyrrhiza glabra*	Glycyrrhizic acid	Pluronic F127/d-a-tocopheryl PEG1000 succinate mixed micelles-based hydrogel (MMs-gel)	The gel was effective in suppressing AD symptoms, including oedema, high IgE levels, skin lesions, and mast cell infiltration, epidermal hyperplasia	Micelle system enhanced solubility and dermal bioavailability	[50]
*Glycyrrhiza glabra*	Glabridin	Topical application in DNFB-induced AD mice	Suppresses activation of NF-κB and MAPK pathways, significantly inflammatory cell infiltration reduced skin thickening, and levels of IFN-γ, TNF-α, IL13, IL6, IL5, IL4, and IgE	Topical route minimized systemic toxicity while maintaining anti-inflammatory activity	[51]
*Aloe*	Aloe gel with olive oil	Combination cream (Olivederma)	Significantly reduced SCORAD severity score (~ 64.5% vs. 13.5% in betamethasone); decreased serum IgE and eosinophil counts; improved quality of life via anti-inflammatory and skin barrier effects	Showed superior clinical improvement vs. betamethasone	[52]
*Aloe*	Aloe	Processed Aloe vera gel given orally to OVA-induced AD mice	Suppressed total and OVA-specific IgE, reduced epidermal thickness, restored tight-junction proteins (ZO-1, Claudin-1/8), lowered IL4 and IL17A in skin	Systemic administration improved immune balance and barrier protein expression, demonstrating multi-targeted efficacy	[53]
*Aloe vera* + *Quercetin*	Aloin + Quercetin	Topical dermal formulation (cream/gel) of Aloe vera + Quercetin	Moisturises skin, enhances skin hydration, barrier improvement; anti-inflammatory and antimicrobial effects	Combination provided complementary antioxidant + barrier-restorative effects, improving skin hydration and lesion resolution	[54]
*Azadirachta indica*	Ethanolic leaf extract	1% topical ointment induced AD-like mice	Reduced erythema, scaling, and edema; improved skin hydration and barrier function; lowered serum IgE and IL4; suggests suppression of inflammatory cytokines and NF-κB-mediated pathways with topical neem treatment	Topical delivery enhanced local anti-inflammatory action with reduced systemic burden	[55]
*Matricaria Chamomile and Bletilla striata*	Bletilla striata polysaccharides and Chamomile volatile oil	Bletilla striata polysaccharide based Nanoemulsion gel	Anti-inflammatory and skin barrier repair by reducing Th2 cytokines (IL4, IL5), inhibiting mast cell infiltration, enhancing filaggrin expression, and repairing skin lesions	Nanoemulsion improved skin penetration and barrier repair, supporting synergistic botanical action	[56]
*Borago officinalis*	γ-Linolenic acid (GLA), tocopherols, phytosterols	-	Antioxidant action through free radical scavenging, lipid peroxidation inhibition; potential anti-inflammatory effects	Natural lipid components support restoration of skin lipid balance	[57]
*Cocos nucifera*	Lauric acid and polyphenols	tested as oil/extract	Decrease pro-inflammatory cytokines (TNF-α, IFN-γ, IL8, IL6, IL5 in THP-1 cells	Lauric acid provides antimicrobial and anti-inflammatory effects while acting as an emollient to reduce TEWL	[58]
*Vitellaria paradoxa*	Methanolic kernel extract	tested as extract in vitro	Decrease LPS-induced NO, IL1β, IL12, TNF-α production;	Rich fatty acid profile supports barrier repair while providing anti-inflammatory antioxidant activity	[59]
*Rosa spp. *[60]	Rose essential oils and phenolic compounds	Topical herbal cream tested in animal model	Improved skin barrier and reduced inflammation; likely decreased erythema and edema in an eczema model	Phenolic antioxidants assist epidermal repair while reducing inflammatory responses	[61]
Honey	Methylglyoxal, flavonoids, phenolic acids	Topical formulation	Decrease progression rate of AD; strong anti-staphylococcal effect, reducing S. aureus–related inflammation in AD	Provides antimicrobial + anti-inflammatory actions simultaneously, targeting both infection and inflammation	[62]
Honey	Honey, Olive Oil and Beeswax	Topical mixture (ointment-like)	Effective in managing AD, likely due to synergistic, emollient effects and anti-inflammatory	Synergistic formulation improved moisturization and barrier function, supporting lesion healing while reducing irritancy risk	[63]
Rosemary	Carnosol	Topical formulation	Decrease IgE, TNF α, IL 1β; Decrease iNOS and COX 2; histology showed reduced epidermal thickness and inflammation	Topical delivery enabled localized cytokine suppression and barrier restoration without systemic side effects	[64]
Quercetin	Quercetin	In vitro keratinocyte model—pre-treatment of HaCaT cells with 1.5 µM quercetin before AD-inducing cytokines (IL-4, IL-13, TNF-α)	Reduced expression of IL-1β, IL-6, IL-8, TSLP (pro-inflammatory cytokines) and increased antioxidant enzymes: SOD1, SOD2, catalase, glutathione peroxidase, and increased IL-10	Demonstrated strong immunomodulatory and antioxidant potential	[65]
Quercetin	Quercetin	Liposomes-in-gel: quercetin-loaded liposomes (QU-L) dispersed in 1% sodium carboxymethyl cellulose (CMC-Na) gel (QU-LG)	Good antioxidant activity (DPPH free radical scavenging ~ 65.16 ± 3.513%) • Inhibition of malondialdehyde (MDA) production in liver and skin (marker of lipid peroxidation) better than dexamethasone cream • Treatment in mouse cutaneous eczema model: significant reduction in dermatopathological symptoms (erythema, oedema) compared to untreated controls	Liposome-in-gel system enhanced stability, dermal uptake, and therapeutic efficacy vs. free quercetin and dexamethasone cream	[66]
β-carotene or lycopene	β-carotene or lycopene	Oral dietary administration (carotenoid supplemented diet) in HR-1 hairless mice fed low zinc/magnesium diet	Reduced AD-like dermatitis severity (clinical & histological) in mice • Decreased epidermal thickening and inflammatory cell infiltration • Suppressed Th2-type chemokines (e.g., CCL27 for β-carotene group; TARC for lycopene group) in skin	Dietary supplementation demonstrated systemic immunomodulation, suggesting supportive adjunct role rather than standalone therapy	[67]
Kaempferol	Kaempferol	Nanoemulsion-based gel: Kaempferol loaded nanoemulsion incorporated into a gel matrix	Improved solubility and skin permeation of kaempferol via nanoemulsion delivery • Enhanced delivery to skin likely reduced erythema via anti-inflammatory/antioxidant effects	Nanoemulsion enhanced kaempferol solubility and skin permeation, improving topical therapeutic response	[68]

### Polymeric conjugated topical preparations for atopic dermatitis

In recent years, the limitations of conventional topical therapies in managing AD such as poor skin penetration, rapid degradation of active ingredients, and variable patient adherence have driven the search for more advanced, targeted approaches. Nanotechnology and polymer-based systems have emerged as promising solutions that can enhance therapeutic efficacy by improving drug solubility, stability, and controlled release. Among these, hydrophilic/hydrophobic polymeric conjugates offer unique advantages for topical applications due to their ability to encapsulate a broad range of active compounds and facilitate their sustained delivery through the compromised skin barrier.

The polymeric conjugates are advanced amphiphilic systems engineered by chemically or physically linking hydrophilic polymers such as polyethylene glycol, polyvinyl alcohol [69, 70], or chitosan [71] with hydrophobic polymers like polylactic acid, polycaprolactone [72], or polydimethylsiloxane. These hybrid polymers can self-assemble into versatile nanostructures such as micelles, nanogels, or thin films, capable of encapsulating both hydrophilic and hydrophobic therapeutic agents within a single system. The amphiphilic nature of these conjugates allows for deep dermal penetration by mimicking the natural hydrophilic-lipophilic balance of the skin. Furthermore, these systems can be engineered to respond to specific pathophysiological stimuli such as acidic pH or elevated enzyme activity enabling controlled, site-specific, and on-demand drug release [73, 74].

In atopic dermatitis, the polymeric conjugates and self-assembled nanocarriers are used to increase therapeutic effects by promoting drug solubility, stability and controlled release and facilitates the penetration of drugs through the highly impaired but still protective stratum corneum. Amphiphilic polymers spontaneously assemble into micelles, nanogels or vesicles containing hydrophobic cores with solubilizing lipophilic anti-inflammatory agents and hydrophilic shells with stabilizing action that reduce irritation—a key requirement for AD's sensitive skin. They ameliorate the hydration and pliability of the stratum corneum layer. They fluidize damaged lipid molecules, which by way of follicular pathways facilitates improved penetration of the skin without compromising barrier integrity. Their ability to adhere to skin, entrap unstable drugs (e.g., corticosteroids, calcineurin inhibitors, and antioxidants), and provide sustained delivery to the skin enhances their effectiveness. Their efficacy lies in decreasing inflammation, itching, and frequency of flares in atopic dermatitis while limiting systemic exposure and local adverse effects. Summary in recent finding associated with use of different polymeric bioconjugates for the management of AD are presented in Table 2.

**Table 2:** Summary in recent finding associated with use of different polymeric bioconjugates for the management of AD

Polymer and its type	Hydrophilic/hydrophobic	Main properties	Advantages	Disadvantages/limitations	Applications in AD	Refs
Hyaluronic Acid (HA)	Hydrophilic	Biocompatible; moisture-retaining; promotes skin hydration and healing	Enhances skin hydration, elasticity; supports barrier repair; biodegradable	Rapid degradation; low mechanical strength	Used in HA hydrogels containing chitosan nanoparticles (CS–TPP) with resveratrol; decreases ROS, TNF-α, and IFN-γ in HaCaT keratinocytes; promotes anti-inflammatory and antioxidant effects	[75, 76]
Chitosan	Hydrophilic (cationic biopolymer)	Biodegradable; antimicrobial; film-forming	Excellent biocompatibility; wound-healing; enhances drug penetration	Poor solubility in neutral pH; limited elasticity	Used in chitosan hydrogels and nanoparticles for antioxidant and anti-inflammatory delivery; hollow MnO₂–chitosan hydrogel suppressed inflammation and scavenged ROS in AD models	[77, 78]
Alginate	Hydrophilic	Anionic polysaccharide from brown algae; forms gels with divalent cations	Non-toxic; good moisture retention; suitable for hydrogels	Brittle gel network; sensitive to ionic environment	Used as hydrogel base in wound and eczema therapy; enhances moisture balance in dry AD lesions	[79, 80]
Poly (glycidyl methacrylate) (PGMA)	Hydrophobic	Synthetic polymer with functional epoxy groups	Modifiable surface; enables crosslinking for controlled drug release	Potential cytotoxicity at high concentrations	Explored in nanocarriers for targeted skin delivery in AD drug systems	[81]
Polycaprolactone (PCL)	Hydrophobic	Biodegradable polyester; flexible; good mechanical strength	Sustained drug release; good compatibility with PEG	Slow degradation rate; limited hydrophilicity	Used in PEG–PCL amphiphilic copolymers to improve drug solubility and dermal permeability	[82]
Poly (lactic-co-glycolic acid) (PLGA)	Amphiphilic (hydrophobic–hydrophilic)	FDA-approved copolymer; tunable degradation; forms nanoparticles	Sustained, controlled drug delivery; biocompatible	Requires organic solvents; may induce acidic degradation products	PLGA–PEG nanoparticles functionalized with RGD peptides and HA improved skin barrier repair and reduced inflammation in AD models	[83]
Polyvinyl Alcohol (PVA)	Hydrophilic	Biocompatible, film-forming, water-soluble	Forms stable microneedles or nanogels; non-toxic	Poor mechanical strength when hydrated	Used in PVA/PVP microneedles for sustained transdermal drug delivery in AD; improved permeability and reduced skin trauma	[84, 85]
Polyethylene Glycol (PEG)	Hydrophilic	Non-ionic polymer; enhances solubility and biocompatibility	Improves skin permeation; non-immunogenic; stabilizes nanoparticles	May cause irritation with chronic exposure	PEG–PCL copolymers enhance dermal penetration, sustain release, reduce irritation in AD	[86, 87]

The development of such amphiphilic polymeric conjugates has emerged as a promising strategy for the topical treatment of AD, a chronic, relapsing skin disease characterized by immune dysregulation, epidermal barrier dysfunction, and microbial imbalance. Conventional therapies including topical corticosteroids, calcineurin inhibitors, and JAK inhibitors while effective, often suffer from limitations such as systemic side effects, poor dermal penetration, and the need for frequent reapplication, reducing long-term compliance and increasing the risk of adverse reactions [88]. To address these limitations, advanced amphiphilic systems combining hydrophilic components like multi-arm PEG with hydrophobic segments such as PDMS or PCL have been synthesized. These conjugates enhance the solubility of poorly water-soluble drugs, improve dermal permeability, and sustain drug release while minimizing systemic absorption. They also offer the potential for co-delivery of multiple therapeutic agents, thus targeting various aspects of AD pathogenesis such as inflammation, oxidative stress, microbial overgrowth, and barrier disruption simultaneously [89, 90].

A notable example is the 4-arm amphiphilic copolymer developed by Jeong et al. (2025), comprising PEG and PDMS. This conjugate was successfully loaded with both hydrophobic deoxycholic acid (DCA) and hydrophilic methylene blue, demonstrating encapsulation efficiencies exceeding 98%, dermal permeation greater than 90%, and excellent biocompatibility in murine models. Repeated topical application did not result in skin irritation, inflammation, or systemic organ toxicity, highlighting the safety and suitability of such formulations for chronic use in AD [89].

Further innovation in this area has led to the development of stimuli-responsive conjugate systems that release therapeutic agents in response to the pathological cues characteristic of AD skin such as elevated protease activity or local acidosis. These systems can be integrated into minimally invasive devices, such as dissolving microneedles, to enhance delivery precision and patient comfort. Recent studies using PVA/PVP-based microneedles have demonstrated significant improvements in transdermal and ocular drug penetration, sustained drug release, and reduced skin trauma, making them highly effective for inflammatory skin and eye disorders. Moreover, the versatility of these polymeric conjugates allows for their use with a broad range of AD-relevant therapeutics, including corticosteroids, calcineurin inhibitors, JAK inhibitors, antioxidants, and antimicrobial agents. Their ability to encapsulate both hydrophilic and hydrophobic molecules also supports combination therapy strategies, which are increasingly being explored to address the multifactorial nature of AD.

Ongoing research is focused on optimizing drug–polymer compatibility, improving stimuli-responsiveness, and enhancing clinical translatability. Emerging approaches involve the co-delivery of biologics, gene modulators, or microbiome-friendly agents, offering the potential to further elevate therapeutic outcomes in chronic or treatment-resistant forms of AD. As these technologies continue to evolve, hydrophilic/hydrophobic polymeric conjugates represent a next-generation drug delivery platform capable of transforming topical dermatologic care. Their intelligent design, biocompatibility, and multi-functional capabilities make them well-suited for safer, more effective, and more patient-friendly treatment strategies in AD and potentially other chronic inflammatory skin conditions [91].

Nanotechnology-based drug delivery systems, such as nanoparticles, liposomes, and nanoemulsions, have shown promise in improving skin penetration, targeted delivery, dose control, and clinical outcomes while reducing side effects. Further robust clinical research is urgently needed to confirm these benefits [92].

**Fig. 3:**
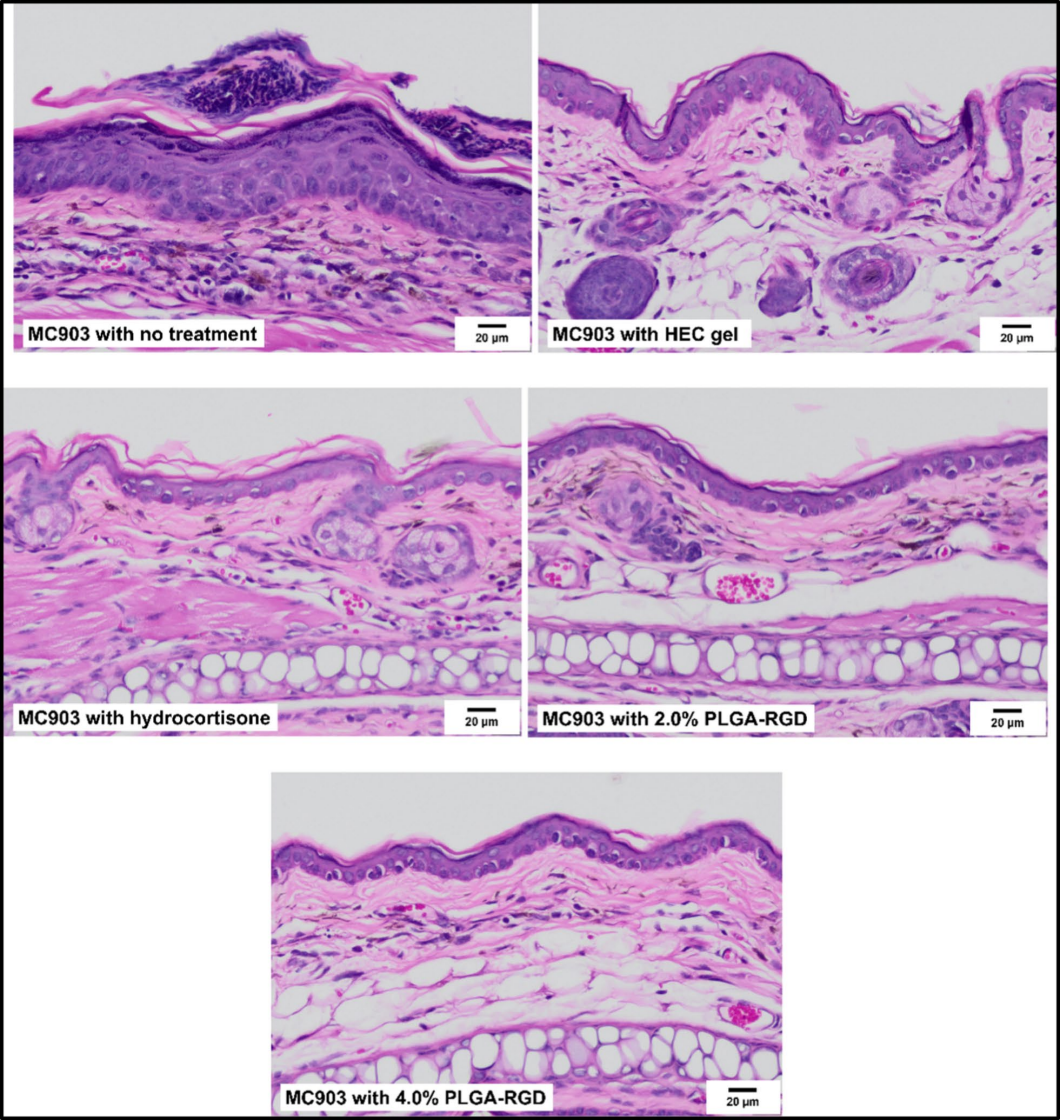
Results of histological analysis from haematoxylin and eosin (H&E) stained mice tissue sections in the in-vivo study with MC903-induced AD animal models (Scale bar = 20 µm). Recreated from [83] under the Creative Commons license NC-ND

**Fig. 4:**
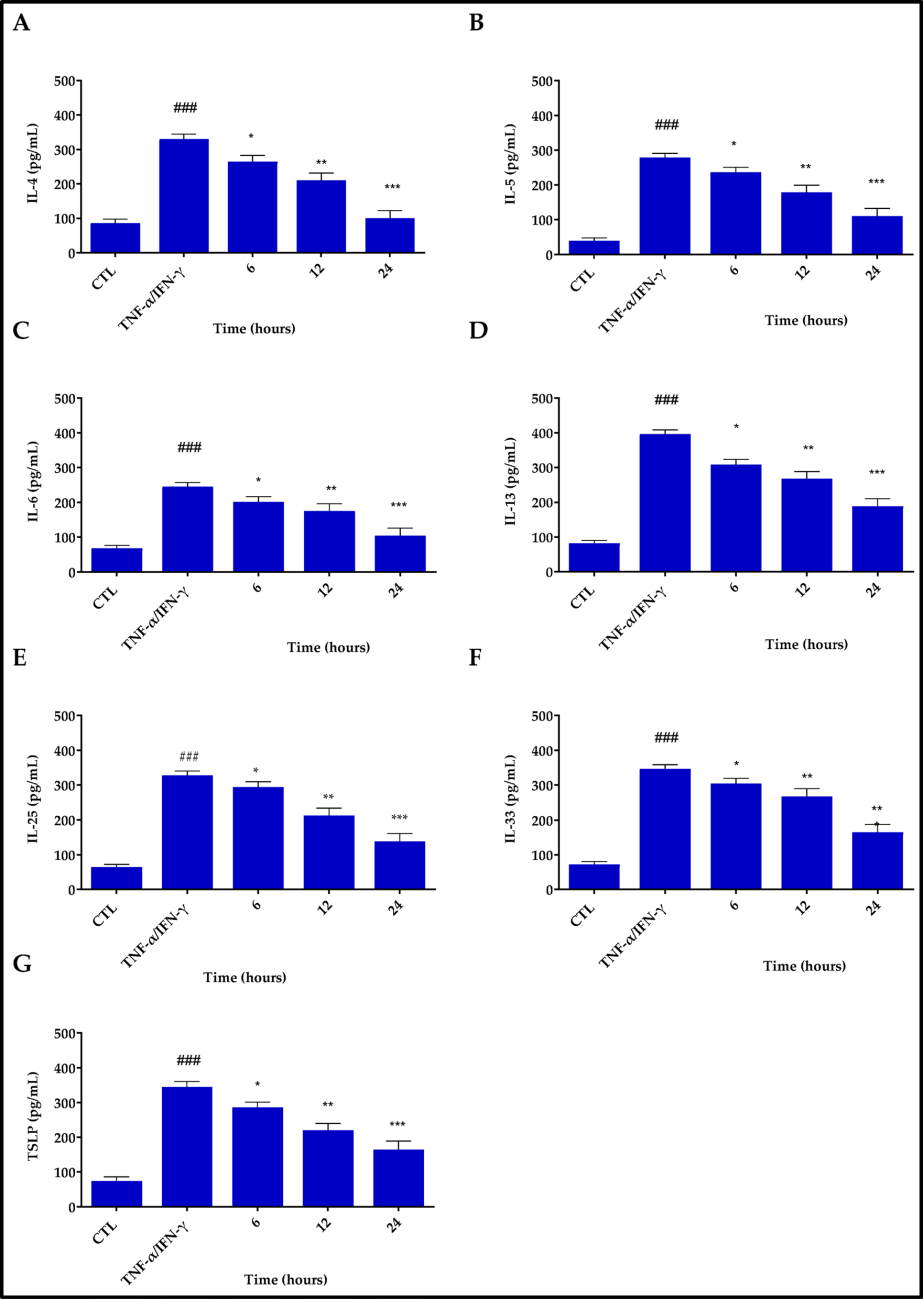
Inhibitory effects of Res@gel10 on the release of inflammatory cytokines in TNF-α/INF-γ-stimulated HaCaT cells. The secretion levels of IL4 **A**, IL5 **B**, IL6 **C**, IL13 **D**, IL25 **E**, IL33 **F**, and TSLP **G** were quantified using an ELISA technique. Cells underwent pre-treatment with Res@gel10 for 24 h, followed by stimulation with TNF-α/IFN-γ for an additional 24 h. Results are shown as the mean of three separate experiments ± standard deviation (n = 3). ### p < 0.001 for TNF-α/IFN-γ-treated cells compared to CTL; * p < 0.05, ** p < 0.01, and *** p < 0.001 for Res@gel10 compared to TNF-α/IFN-γ-treated cells. Recreated from [75] under Creative Commons licence 4.0

Park et al. (2025) engineered amphiphilic poly(lactic-co-glycolic acid)-b-poly(ethylene glycol) (PLGA-PEG) copolymers functionalized with bioactive ligands such as RGD peptides and hyaluronic acid using ring-opening metathesis polymerization. The resulting nanoparticles (~ 150 nm) were incorporated into a hydroxyethyl cellulose gel and demonstrated excellent biocompatibility in 3D human skin models. In an MC903-induced AD mouse model, the RGD-conjugated gel significantly reduced inflammation and accelerated skin barrier repair, outperforming hydrocortisone. The optimized nanoparticle size enhanced skin penetration and surface interaction, highlighting the therapeutic potential of peptide-functionalized PLGA-based nanocarriers for targeted AD treatment [83] (Fig. 3**)**. Conte et al. (2023) developed a hyaluronic acid (HA) hydrogel embedded with chitosan–tripolyphosphate (CS–TPP) nanoparticles containing ~ 80% encapsulated resveratrol (~ 120–500 nm in diameter). Nanoprecipitation and ionic gelation were used to formulate the nanoparticles, which were then dispersed in an HA network. This composite hydrogel exhibited sustained release, resisted enzymatic degradation, and, in vitro, significantly decreased ROS-induced proinflammatory cytokines (TNF-α, IFN-γ) in HaCaT keratinocytes. The study highlighted its potential as a stable antioxidant/anti-inflammatory topical for AD [75] (Fig. 4**)**.

**Fig. 5:**
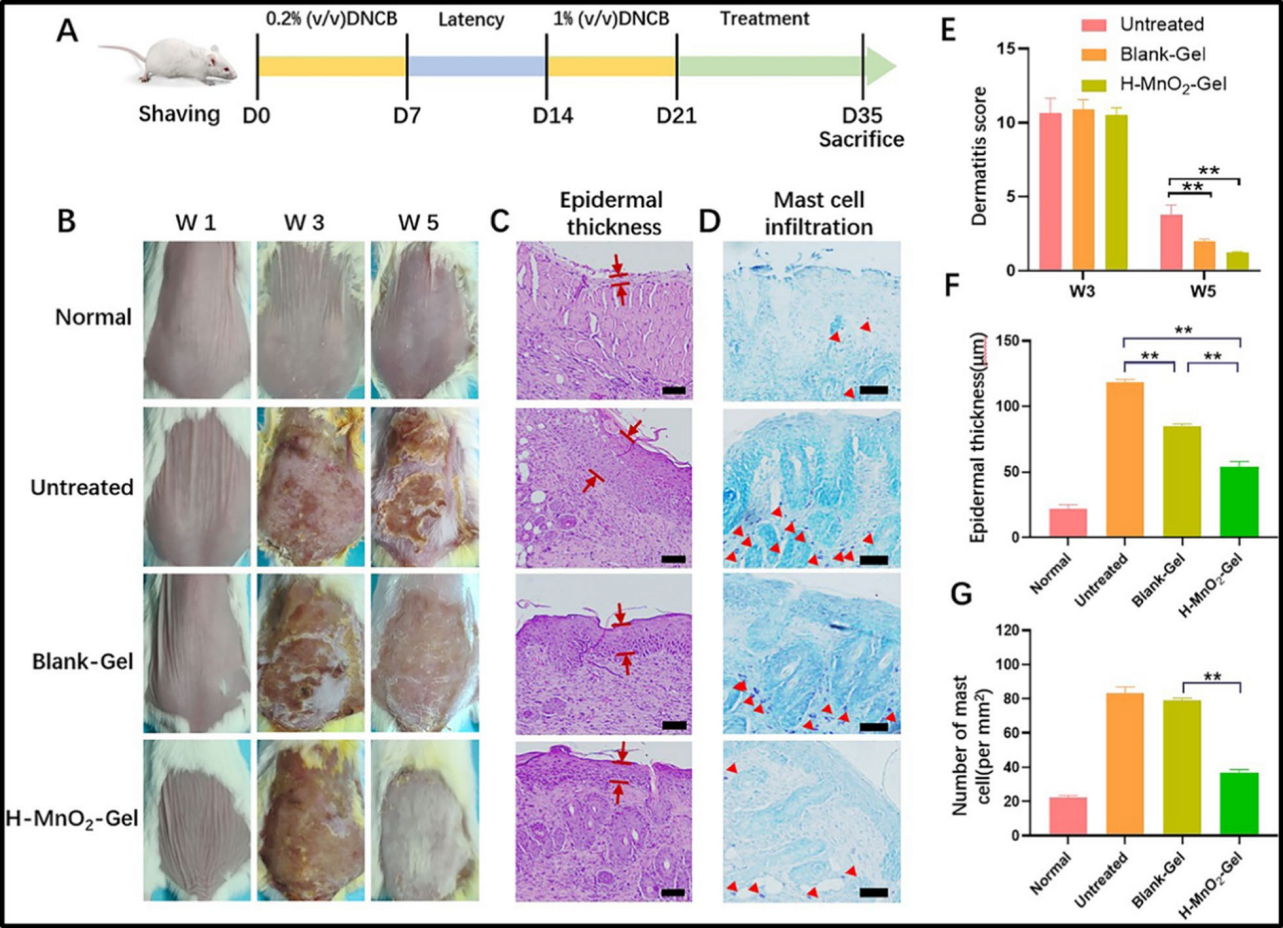
Schedule for executing in vivo research on disease induction by sensitisation with DNCB and subsequent interventions using hydrogel patches **A**. Representative images of the dorsal skin from each group to observe changes in the lesion **B**. Histological examination of mouse dermal tissue slices stained with haematoxylin and eosin. The interval between the red lines denotes the measurement of epidermal thickness **C**. Histological analysis of murine skin tissue slices stained with toluidine blue to detect dermal mast cells. The mast cell cells are marked by red arrowheads (scale bar = 100 μm) **D**. Dermatitis score assessments conducted over a period of 5 weeks (E). Measure of epidermal thickness **F**. Assessment of mast cell quantity for each therapy cohort. Sample size (n = 6). **P < 0.01 **G**. Recreated from [77] under Creative Commons licence 4.0

Slavkova et al. (2024) prepared Eudragit L100 nanoparticles (57 nm, − 31 mV, ~ 90% encapsulation) loaded with budesonide via nanoprecipitation. These were suspended in methylcellulose and Pluronic F127 hydrogels. Characterization included rheology, occlusion, human keratinocyte safety tests, and ex vivo permeation. Results revealed controlled corticosteroid release, efficient skin penetration, and reduced systemic exposure, suggesting a safer paediatric AD treatment option [93]. Wu et al. (2023) created a quaternary chitosan (QCS)/tannic acid gel crosslinked with hollow MnO₂ nanoparticles. Utilizing dynamic ionic hydrogen bonding, the hydrogel showed strong oxidative stress scavenging and moisture retention. In AD mice, it significantly reduced epidermal thickness, mast cell infiltration, and serum IgE. The study highlighted its dual abilities: ROS clearance and immunomodulation, supporting its promise in AD therapy [77] (Fig. 5). Abdelghany et al. (2019) formulated curcumin nanosuspensions (CU-NS) via nanoprecipitation (520 nm), embedded into poly (vinyl alcohol) (PVA) microneedle arrays (900 µm height). These microneedles withstood 32 N, penetrated porcine skin, dissolved within 60 min, and delivered curcumin to ~ 2300 µm depth 6 × deeper than topical application. The system promotes effective intradermal delivery of hydrophobic actives, a notable advance in AD drug delivery [4].

**Fig. 6:**
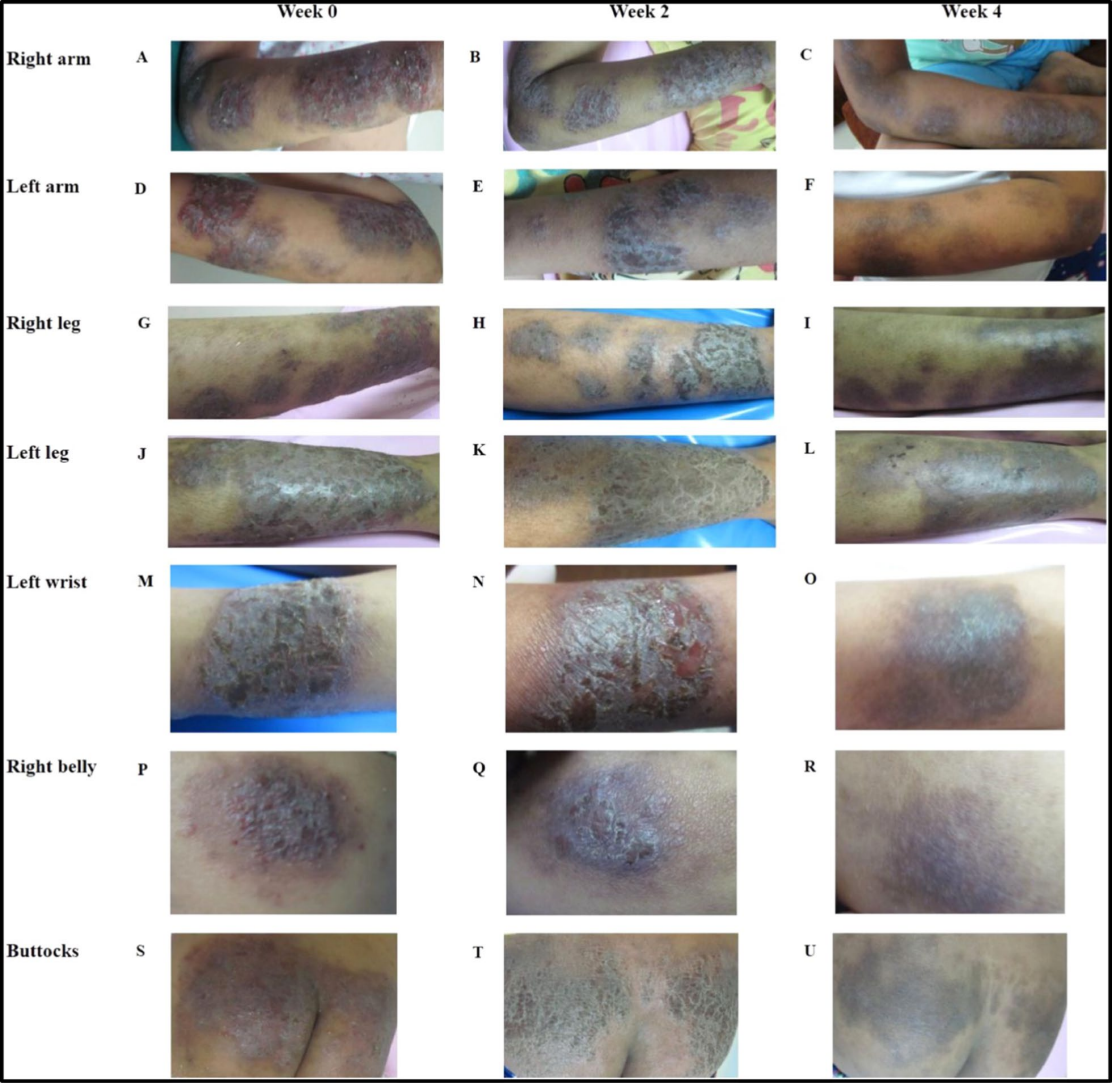
Traditional Thai based-herbal preparation were used to treat skin lesions, which resulted in clinical improvement. Baseline photos from week 0 revealed the status of the right arm **A**, left arm (**D**), right leg (**G**), left leg (**J**), left wrist (**M**), right abdomen (**P**), and buttocks (**S**). Subsequent images taken two and four weeks after treatment demonstrated considerable improvement in skin condition over the same regions: right arm (**B**, **C**), left arm (**E**, **F**), right leg (**H**, **I**), left leg (**K**, **L**), left wrist (**N**, **O**), right belly (**Q**, **R**), and buttocks (**T**, **U**). These photos show a considerable reduction in lesion severity over the therapy period. Recreated with permission from [97] Right Link Share Copyright Clearance Center

**Fig. 7:**
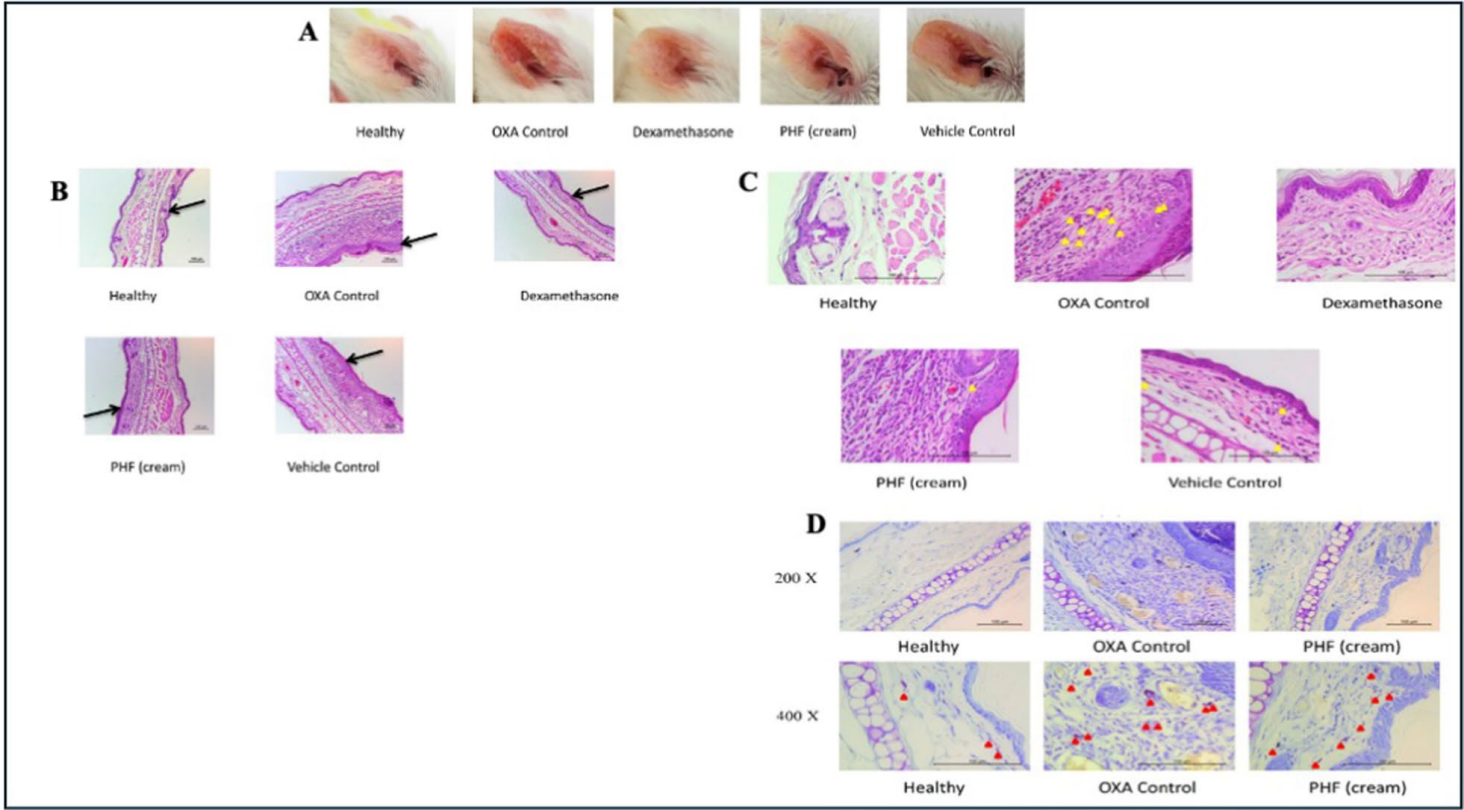
Images of ear redness in mice treated to various treatments were recorded on Day 27, right before sacrifice (n = 6) **A**. On Day 32, H&E-stained slices of the epidermis and dermis from treated (right) ears were collected at 100 × magnification to examine tissue morphology under various treatment conditions **B**. On Day 32, H&E-stained slices from the same samples were examined at a greater magnification (400 ×). Yellow arrows indicate eosinophil infiltration in the dermal layer **C**. To visualize mast cell infiltration, ear tissue was stained with toluidine blue at 200 × and 400 × magnifications on Day 32. Red arrows indicate mast cell infiltration in the dermal region **D**. Recreated with permission from [100] under Creative Commons licence 4.0

A hydrogel-based microneedle patch has been developed to control transdermal delivery of cyclosporine for treating AD. The patch demonstrates excellent biocompatibility, ROS scavenging, bacterial eradication, and ROS-responsive drug release capabilities. In a mouse model, It exhibited better benefits at 29% of the oral dosage rendering it a promising treatment approach for alleviating AD pathogenesis [94]. Yan et al. (2024) synthesized temperature- and enzyme-responsive poly (N isopropylacrylamide) (PNIPAAm) hydrogels loaded with a combination of betamethasone dipropionate (BDP) and halometasone (HE). Polymerization involved free radical methods with crosslinkers. Upon topical application, these hydrogels released > 80% drug content over 72 h in conditions simulating AD skin, achieving sustained dual-therapy delivery, potentially reducing dosing frequency and systemic exposure [95]. Recent research used traditional Thai herbal medicine on the infected region of an enrolled patient, (an 8 year girl with persistent eczema), daily, including a lymphatic skin therapy. and haematic tonic therapy. This drug was supplemented with two external formulations, such as bath preparation and topical treatment, for four-five weeks. The results demonstrated reduced score in itching and dark-thinner skin lesions after two weeks of application of medications. Additionally, during the fourth week of tests, all skin lesions had entirely disappeared, leaving only minor post-inflammatory pigmentation. Furthermore, follow-up examination after three months, no recurrence was observed. These results suggested that combination of bioactive rich formulations can be used for successful management of chronic eczema that come under category of AD [96]. In a similar report on use of Penta herbs formula, which is categorised under traditional Chinese medicine for the management of AD. The Penta herbs formula containing *Cortex Moutan,* root bark of *Paeonia suffruticosa Andr. (from Ranunculaceae), Cortex Phellodendri, bark of Phellodendron chinensis Schneid (from Rutaceae), Flos Lonicerae,* flower of *Lonicera japonica Thunb. (from Capri-foliaceae), Herba Menthae,* aerial part of *Mentha Haplocalyx Briq. (Labiatae)* and *Rhizoma Atractylodis,* and rhizome of *Atractylodes lancea* (Thumb.) DC. (from Compositae) at a ratio of 2:2:2:1:2 was used for the treatment of eczema. The results suggested that, though individual herbs showed modulation of mast cells differently such as attenuation of histamine release and prostaglandin D2 synthesis, suppression of mediator release, the suppression of inflammatory mediator release from cells called mast cells may have contributed to the effectiveness of therapy of Penta herbs formula [97] (Fig. 6). Similar results were reported by Kirby and Schmidt for Chinese herb in the effective management of chronic AD via placebo controlled trails [98].While, Park and co-worker reported that application of bioactive compound-based formulation significantly suppressed the AD-like skin lesions when tested over 2,4-dinitrochlorobenzene in the NC/Nga murine model [99]. Moreover, Tsang and co-workers studied the anti-inflammatory activities of Penta herbs incorporated cream followed with efficacy on in vivo murine model with oxazolone-mediated dermatitis. The results demonstrated a significant reduction in dermatitis upon application of Penta herbs incorporated cream might be due to presence of gallic acid, chlorogenic acid, and berberine. The presence of these bioactive compounds further showed significant reduction of pro-inflammatory cytokines (IL6) and chemokine, compared to dexamethasone, suggesting potential use of Penta herbs incorporated cream in management of AD [100] (Fig. 7).

**Fig. 8:**
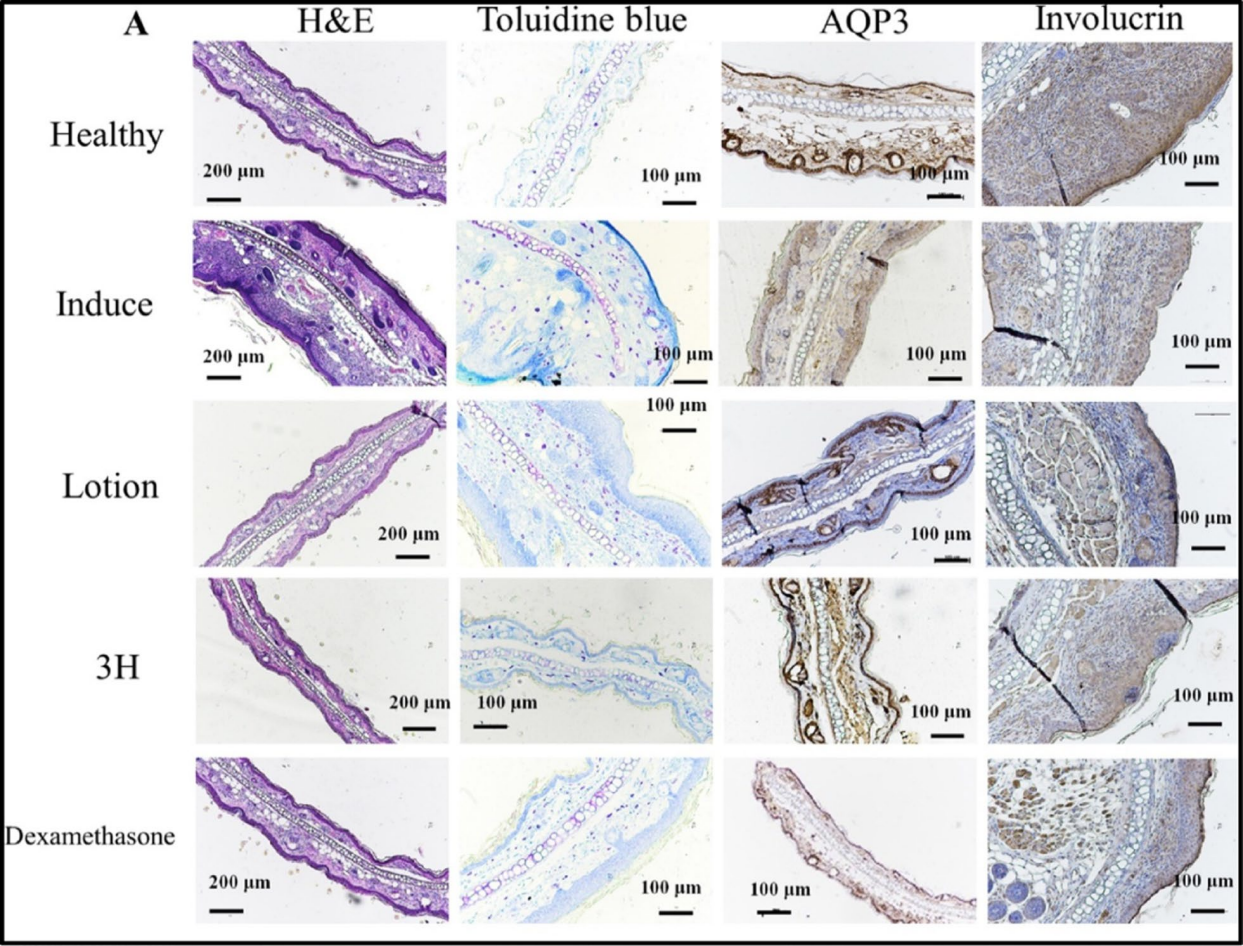
Different types of stains on mice ears that have AD-like symptoms from oxazolone tissue. (A) Representative photographs of H&E, toluidine blue, AQP3 and involucrin staining of challenged ear (right ear) upon different treatments (100 ×). Reproduced with permission from [101] RightLink Share CopyRight Clearance Center

Wu et.al., (2021) tested herbal formulations containing *Herba Menthae* and*, Cortex Moutan* with *C. officinalis* at ratio of 1:1:1 in fortified cream formulation topically. The findings indicated a significant reduction in the synthesis of IL6 and tumour necrosis factor (TNF)-α in HMC-1 cells, reduced the expression of IL6, IL8, and CCL2 in TNF-α/INF-γ stimulated HaCaT cells, and curtailed LPS-induced nitric oxide generation in macrophage cells. Additionally, in vivo experimental study demonstrated reduction in ear swelling and scratching frequencies. Moreover, the H&E and toluidine staining indicated reduction in epidermal thickness and mast cell infiltrations, suggesting potential role of bioactive compounds in management of AD [101] (Fig. 8).

Iraji et.al., (2024) reported the effectiveness of an herbal formulation (cream) constituting of *Glycyrrhiza glabra Curcuma longa, Calendula officinalis,* and *Fumaria parviflora*, compared with mometaone 0.01% topical formulation in the management of AD. The results of clinical outcome suggested a significant high scores (SCORAD system) for patient who receive the herbal composition than those received standard formulation (cream). Although the overall results indicated a lower efficacy of herbal combinations, compared to mometasone, a low associated complication with herbal formulation could promising in the management of AD [102]. Additionally, study conducted on 40 patients with mild to moderate eczema, who randomly received mometasone (0.1%) or herbal cream composed of *Silybum marianum* and *Fumaria officinalis* demonstrated significant reduction in SCORAD system-based score for both treated groups. Interestingly the overall score suggested that cream fortified with herbs can reduce the severity and symptoms of AD similar to standard treated formulation, thus tested plant can be considered as a new treatment strategy in management of eczema symptoms and associated complications [102]. A formulation Derma-Hc constituting of *Schizonepeta tenuifolia, Atragalus membrabaceus Cryptotympana pustulata, Angelica sinensis,* and *Arctium lappa* was tested on TNF-α and skin lesions with IFN-γ-treated with HaCaT including keratinocyte differentiation genes and assessment of signalling pathway. The results demonstrated that bioactive compound fortified formulations markedly inhibited the epidermal hyperplasia, hyperkeratosis, and mast cells infiltration. While the formulation inhibited several factor associated with AD, it restored expression level of APINK5 and inhibited IL22 via the blockade of JAK1-STAT3 signalling pathway. These results suggested the combination of herbs rich in bioactive components can be promising in management of AD [103].

Khalighi et. al., fabricated liposomal incorporated formulation fortified with *Althaea officinalis* flower extract for the management of AD and compared the efficacy against steroids through double-blind controlled trail phase II, on 40 patients who exhibit moderated allergic AD, which was confirmed by skin prick test. Based on SCORAD score the results suggested that liposomal formulations significantly reduced the conditions. This was further confirmed on comparing the score on two weeks and four weeks were score was lower on left side for treatment with steroids and lower on both side with insignificant difference and no side effect between test and standard. These results suggested higher potential of *Althaea officinalis* flower extract in management of AD [104]

### Clinical trials and patents information

The treatment of AD is rapidly evolving, with a focus on targeted immunotherapies, small molecules, and alternative formulations like herbal and nanotechnology-enhanced topicals. Clinical trials are ongoing globally to assess the safety, efficacy, and patient-centered outcomes of these emerging therapies. These trials reflect a shift towards individualized and mechanism-based approaches in AD management, as well as increasing interest in non-steroidal and complementary therapies aimed at improving long-term disease control, minimizing adverse effects, and enhancing quality of life. Numerous recent developments in topical delivery methods for the treatment of AD are covered by both national and international patents. These technologies include hydrogels, nanoemulsions, polymeric carriers, and immunomodulatory formulations.

**Fig. 9:**
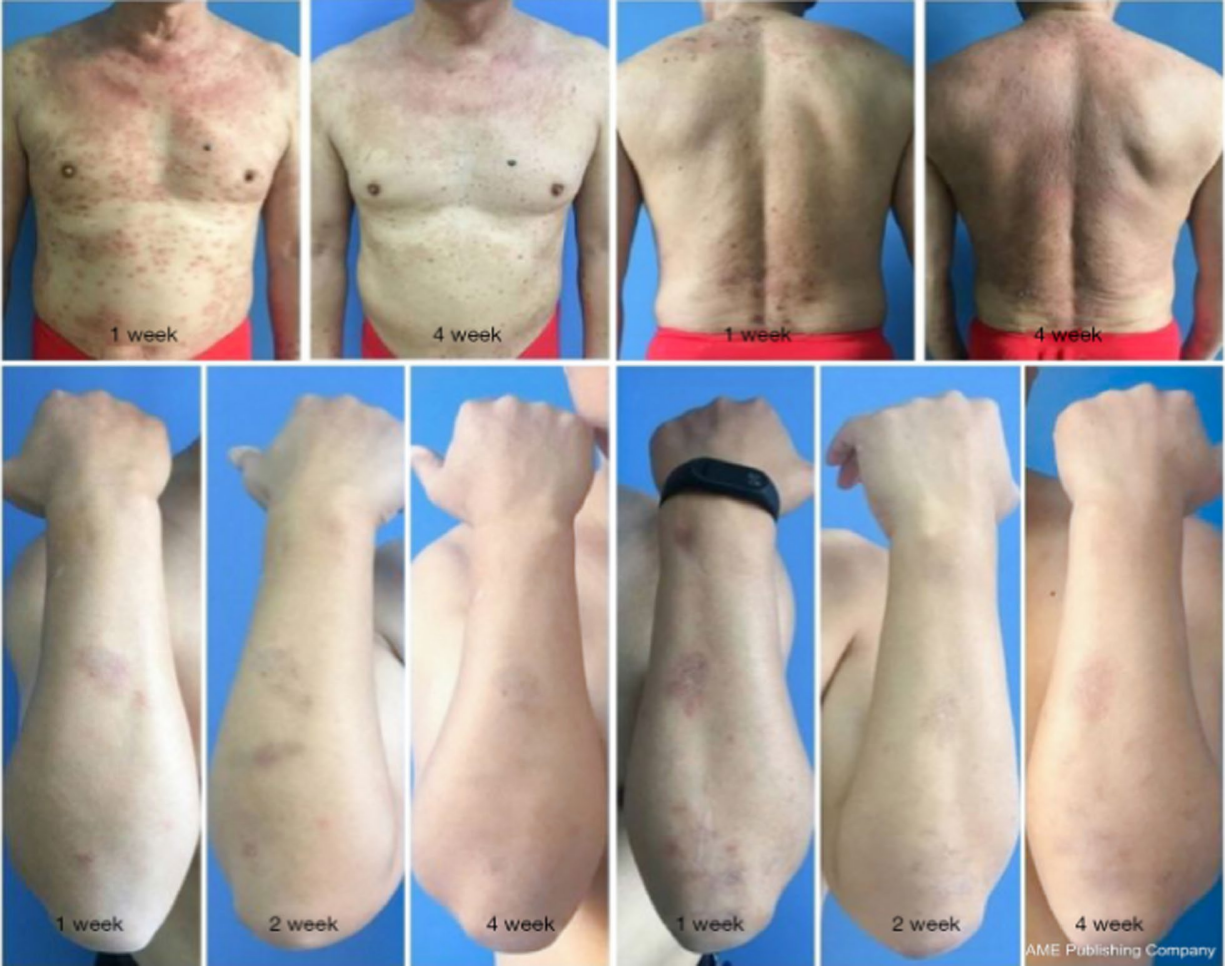
Patients administered Qinzhuliangxue exhibited significant improvement post-treatment, resulting in a notable reduction of erythema, reduction of swelling, and antipruritic effects. Recreated with permission from [105] under Creative Commons Attribution-NonCommercial-NoDerivs 4.0 International License (CC BY-NC-ND 4.0)

**Fig. 10:**
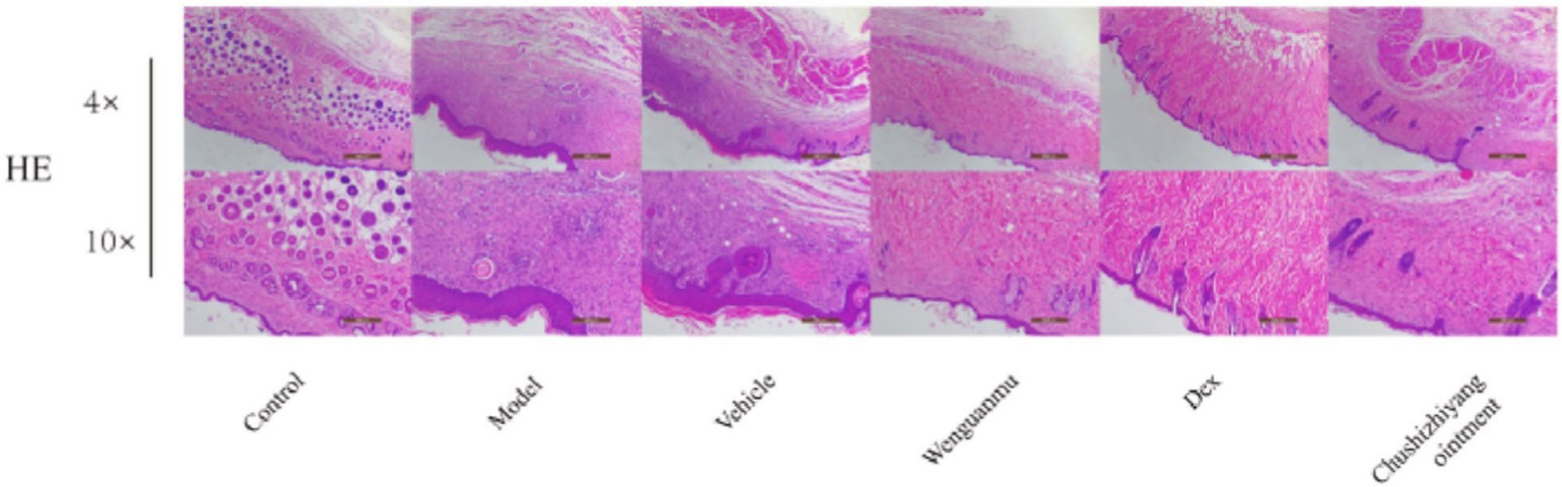
Illustration showing effect of Wenguanmu ointment on eczema model mice; where HE staining of the back skin showing the thickness of the dorsal basal cell layer of the model group increased, and at the same time, the inflammatory cell infiltration in the dorsal skin tissue of eczema-like increased. Reproduced with permission from [107], RightLink Share CopyRight Clearance Center

A clinical trial conducted on efficacy and safety of Qinzhuliangxue in management of AD over 176 patients revealed score of X^2^ = 14.181, recurrence rate of X^2^ = 7.393 and itching score of F = − 3.427. Although the incidence of AD was similar in test and control group, the scores were lower than control group, suggesting bioactive compound-based formulation can be recommended for the management of subacute AD [105] **(**Fig. 9**)**. In another study *Echinacea Purpurea-*derived alkyl amides were tested for the its effect on poly-(I:C) induced pro-inflammatory cytokine expression and release of HaCaT cells. Moreover, irritation and sensitization potential was tested via patch test (clinical trial 1) and clinical efficiency in llevating the AD was tested as clinical trial-2 followed with human skin structure and lipid content in clinical trial-3. The overall results suggested that *Echinacea Purpurea* exhibit potent anti-inflammatory actions by alleviating cutaneous symptoms of AD and ultimately restoring the epidermal lipid barrier [106].Whereas, Zhu and coworker incorporated herbs such as *Xanthoceras sorbifolia* Bunge, *Coptis chinensis* Franch, and *Bezoar* incorporated ointment on eczema mice model. The findings showed that ointment with bioactive substances stops the rise of various pro-inflammatory cytokines and chemokines. It also lowers the synthesis of CKLF-1 as well as NF-κB protein in the cell nucleus and raises the production of IκB protein to help AD. Furthermore, HE stains of the dorsal skin indicate an increase in the thickness of the basal cell layer in the model group, with increased infiltration of inflammatory cells in the eczema-like dorsal skin tissue [107] (Fig. 10). Xuan and co-worker demonstrated the efficacy of Chinese herbal formula Huoxiang Zhengqui in management of AD through double-blinded randomized controlled trial. The study performed for 4 weeks with follow-up on a total of 218 participant following inclusion/exclusion criteria and outcome was measured over eczema area and severity index followed with numerical rating scale, investigator global assessment index, body surface area, skin index-29, and EQ-5D-5L scores, compared to baseline. Although the study didn’t publish the overall output, however it was indicated that positive effect of treatment is [108]. Similar research examined Chinese herbs such Pei Tu Qing Xin granules on kids with mild to severe AD between the ages of 6 and 16. For this investigation, thirty patients participated in a twelve-week treatment period followed by a four-week follow-up as part of a randomised, double-blind, placebo-controlled, parallel clinical trial. Although the study didn’t publish the overall output, however it was indicated that positive effect of treatment is expected [109]. The Tables 3 and 4 lists patents demonstrating the advancement of technology and worldwide research in the field of AD treatment.

**Table 3:** Details of current status of clinical trials for atopic dermatitis

NCT No	Study title	Status	Phase	Interventions
NCT06855745	Phase 2 randomized double-blind study to assess topical zabalafin hydrogel vs vehicle in mild to moderate AD (CLEAR-AD1)	Recruiting	Phase 2 (interventional)	Zabalafin hydrogel, vehicle
NCT06988605	Real-world experience using nemolizumab in the treatment of moderate-to-severe atopic dermatitis in adolescents & adults (RE-UNITE AD)	Not yet recruiting	Observational, phase not applicable	Nemolizumab
NCT06723405	Efficacy study of EVO301 in moderate to severe atopic dermatitis	Recruiting	Phase 2a interventional	EVO301
NCT06436183	A study of the effects of camoteskimab in adults With moderate to severe atopic dermatitis	Active, not recruiting	Phase 2a interventional	Camoteskimab
NCT05642208	Dupilumab step-down strategy to maintain remission in adult and adolescents patients with atopic dermatitis (MADULO)	Recruiting	Phase 4 interventional	Dupilumab (step-down dosing)
NCT06850311	Herbal ointment in treating atopic dermatitis topically	Recruiting	Phase 2/3 study	Sophora flavescens Aiton herbal ointment, Placebo
NCT06855745	Randomized double-blind study to assess topical Zabalafin hydrogel vs vehicle in mild to moderate AD (CLEAR-AD1)	Recruiting	Phase 2, Interventional	Zabalafin Hydrogel, Vehicle
NCT06727552	A Study of barzolvolimab in patients with atopic dermatitis	Recruiting (Active)	Barzolvolimab (150 mg or 300 mg Q4W), Placebo	Randomized, double blind, placebo controlled
NCT00076375	Preliminary study of safety and efficacy of nanocrystalline silver cream in atopic dermatitis (Eczema)	Completed	Interventional, Phase 2	Nanocrystalline Silver Cream
NCT05873933	An investigation of a multi-function skincare product to improve aging, eczema, and acne outcomes	Recruiting	Not specified likely, early phase interventional	Topical emollient containing borage seed oil
NCT05984420	Comparison of limpiAD cream 2.5% plus versus Its vehicle and a basic emollient in patients with atopic dermatitis	Not yet recruiting	Not specified, likely interventional	LimpiAD cream 2.5%, vehicle, basic emollient
NCT06453512	A study to evaluate the emollient performance of doublebase once in the treatment of atopic eczema	Recruiting	Not specified, likely interventional	Doublebase once emollient gel

**Table 4:** Details of current status of patents for atopic dermatitis

Patent number	Inventors/assignee	Description
WO2024176175A1 (2024)	N. BuILBruna, R. A. Scott and I. J. Uings	Methods for treating atopic dermatitis, possibly including use of IL18/IL13 antagonists
WO2024209073A1 (2024)	Foguet M, Gwarek M, and Möllmann L	Pressurized nanoemulsion formulation improving tacrolimus stability and skin penetration
WO2024253345A1 (2024)	ASFAM, Gonçalves de Brito and M. Won Byung-Mook	Hydrogel composition containing natural sugars for topical use in atopic dermatitis
US11241385B2 (2022)	Vyne Therapeutics/Tamarkin et al.)	OILin-water nanoemulsion with narrowly sized droplets (~ 52 nm) containing behentrimonium chloride, glycerin, and cetyl alcohol, designed to treat AD and inhibit *Staphylococcus aureus* biofilms
US 11202814B2 (2021)	M. Mourelle, A. Vasconcelos, R. Galatola	Compositions, microcapsules, or nanocapsules designed for prevention or treatment of atopic dermatitis, pruritus, and atopic eczema
US10201490B2 (2019)	Federico (Polichem Srl)	Chitosan-based nail lacquer composition formulated to treat nail‑associated dermatitis
US10226483B2 (2019)	Doxey R.; Bao J. (Novan Inc.)	Topical compositions combining hydrophilic and hydrophobic polymers (e.g. chitosan, NO‑releasing agents) for skin inflammation including AD
US8636982B2 (2014)	Tamarkin DOV. et al. (Vyne Therapeutics Inc.)	A wax foamable vehicle containing chitosan polymers for treating skin disorders including AD
US10111835B2 (2018)	Maite et al. (Centro Nacional De Tecnología…)	Casein–chitosan microparticle formulation encapsulating probiotics for allergy‑associated diseases including AD
EP1889608B1 (2007)	Nho Y. C. et al. (Korea Atomic Energy Research Institute)	A therapeutic hydrogel composed of chitosan polymer, polyalcohol, and medicinal plant extracts to prevent inflammatory progression in AD

The current trends in clinical trials and AD patent indicate a distinct trend towards more patient-centred, safe, and targeted treatment. Recent trends emphasize the increased use of biologics and small-molecule inhibitors exploiting individual immune pathways, including IL-4, IL-13, and JAK/STAT, as well as there is a growing interest in herbal, bioactive, and nanotechnology-based formulations aimed at reducing side effects and improving skin delivery. Innovation in topical delivery systems also stands out as a particular area where there is innovation; nanoemulsions, polymeric carriers, hydrogels, all extensively backed by a growing number of patents worldwide. Nevertheless, there are significant gaps such as inadequate long-term and large-scale clinical evidence, absence of standardization of herbal preparations, and less comparative or combination trials that combine conventional and natural therapies. Also, there are obstacles such as regulatory barriers, expensive rates and slowness of transferring patented technologies to clinical application. In general, clinical and patent trends are orienting to the customization of treatment, taking a focus on mechanisms, and the absence of steroids, although stronger validation and standardization is needed to make treatment accessible globally and reliable in therapeutic efficacy.

## Challenges and future perspectives

AD, as a clinical problem is still in a state of unresolved clinical dilemma because of its multifactorial pathogenesis, high rate of reappearing the disease even with seemingly successful therapy. Although topical, systemic and biologic therapies have made progress, about 80 percent of children and 60 percent of adults have relapse of the disease in less than one year after stopping treatment, a fact that underscores the chronic relapsing nature of AD [110]. Combination of genetic predisposition, immune mis-regulation and environmental stimuli has remained to make the disease more difficult to control whereas compromised skin barrier integrity has remained to maintain the vulnerability to allergens and colonization with microbes. Existing standard therapy, such as emollients, corticosteroids and calcineurin inhibitors, provide symptomatic relief but do not always provide sustained remission. The safety issues associated with the long-term use of such agents include skin atrophy, tachyphylaxis, and decreased patient compliance, but more recent biologics and JAK inhibitors, despite their promise, are expensive, and there is the concern of systemic immunosuppression and accessibility [110].

The recurrence of AD highlights the key unmet needs, lack of reliable predictive biomarkers of relapse, inter-patient variability of the therapeutic response, and insufficient knowledge of disease endotypes. In addition, psychosocial agents including stress, sleep disorder, and comorbid allergic conditions enhance the burden of the diseases but are not well treated in the daily practices. Further studies should hence focus on precision medicine modalities, i.e. using genomics, proteomics and metabolomics to identify molecular signatures that predict relapse and response to treatment. Next-generation barrier-repair therapies that consist of bioactive compounds, nanocarriers, and microbiome-friendly formulations to reestablish long-term skin homeostasis are also in demand. Digital health solutions to track diseases in real time, monitor adherence, and tele-dermatology can be also implemented to improve proactive management. Therefore, a paradigm shift, which accepts individualized, preventive, and integrative care, is essential. This includes maintenance therapy regardless of remission, early barriers-restoration treatment, patient-focused education, and the integration of pharmacologic and lifestyle treatment like stress relief and nutritional support. The gap between innovation and implementation needs to be closed by collaborative clinical trials which compare multi-modes therapies and real-world outcome research. Introducing molecular understanding into patient-centred care, the future of AD management is able to shift away from symptomatic management to attain lasting remission and enhanced quality of life.

## Conclusions

The management of AD presents a complex challenge due to its chronic nature, recurrence, and diverse aetiology. Synthetic drugs, including corticosteroids, calcineurin inhibitors, and biologics, offer symptomatic relief; nevertheless, their prolonged use is limited by systemic toxicity, localised side effects, and elevated costs. This has stimulated an increasing interest in bioactive chemicals derived from herbal sources, which provide multi-targeted effects with enhanced safety profiles. Integrating these natural agents with sophisticated hydrophilic/hydrophobic polymeric delivery systems has shown considerable promise in surmounting obstacles in topical medication distribution, improving therapeutic efficacy, and reducing unwanted effects. Subsequent investigations must emphasise clinical validation, scalability, and regulatory standardisation of these innovative systems. The combined use of natural bioactive compounds with advanced delivery systems may transform the treatment paradigm for AD, providing tailored, efficient, and sustainable management options.

## Data Availability

Dataset can be available from corresponding authors on reasonable request.
